# Evaluating Community Resilience and Associated Factors One Year after the Catastrophic Fort McMurray Flood

**DOI:** 10.3390/ijerph192316153

**Published:** 2022-12-02

**Authors:** Gloria Obuobi-Donkor, Ejemai Eboreime, Reham Shalaby, Belinda Agyapong, Medard K. Adu, Ernest Owusu, Wanying Mao, Folajinmi Oluwasina, Hannah Pazderka, Vincent I. O. Agyapong

**Affiliations:** 1 Department of Psychiatry, Dalhousie University, Halifax, NS B3H 4R2, Canada; 2 Department of Psychiatry, University of Alberta, Edmonton, AB T6G 2R3, Canada

**Keywords:** natural disaster, flooding, mental health, resilience, Fort McMurray, support

## Abstract

Background: Resilience after natural disasters is becoming an increasingly key area of research. In April 2020, parts of Fort McMurray were affected by severe floods. The flooding caused the loss of properties, evacuation of some residents, and effects on their mental health. Objective: This study explores the prevalence and associated factors between flood experience and low resilience a year after the 2020 floods in Fort McMurray. Method: Data collection was accomplished one year after the flood, from 24 April to 2 June 2021, using an online survey. The data were analyzed with SPSS version 25 using univariate analysis with the chi-squared test and binary logistic regression analysis. Results: The prevalence of low resilience was 37.4%. Respondents under 25 years were nearly 26 times more likely to show low resilience (OR = 0.038; 95% CI 0.004–0.384) than respondents 40 years and above. Responders with a history of depression (OR = 0.258 95% CI: 0.089–0.744) and a history of anxiety (OR = 0.212; CI 95% 0.068–0.661) were nearly four to five times more likely to show low resilience than those without a history. Similarly, respondents willing to receive mental health counselling (OR = 0.134 95% CI: 0.047–0.378) were 7.5 times more likely to show low resilience. Participants residing in the same house before the flood were almost 11 times more likely to show low resilience (OR = 0.095; 95% CI 0.021–0.427) than those who relocated. Participants who received support from the Government of Alberta were less likely to express low resilience than those who received no or limited support (OR = 208.343; 95% CI 3.284–13,218.663). Conclusion: The study showed a low resilience rate among respondents following the 2020 flooding in Fort McMurray. Factors contributing to low resilience include age, history of depression or anxiety, and place of residence after the flood. After the flood, receiving support from the government was shown to be a protective factor. Further studies are needed to explore robust risk factors of low resilience and measures to promote normal to high resilience among flood victims in affected communities.

## 1. Introduction

Natural disasters are large-scale events that are often unexpected and, among other things, cause trauma and the destruction of property [1,2]. The Fort McMurray 2020 flooding had a wide-range impact on residents. Hundreds to thousands of homes, businesses, and properties were destroyed, necessitating a large evacuation of residents in the affected vicinity.

Exposure to natural disasters is relatively common, and the individual who is exposed may battle mental health conditions such as post-traumatic stress disorder (PTSD), substance use disorders, anxiety, and depression [3,4]. In Canada, about 12.4 million people experience a disaster in their lifetime, and 73% of this population report a significant disruption to their work and home life [3,5].

The ability to continue functioning after a traumatic event is usually a characteristic of successful adaptation and coping [4,6]. As research advances, attention is now drawn to individual resilience after a traumatic event [7]. The definition of resilience is usually based on two key concepts: adversity and positive adaptation [7,8], which is defined as maintaining a healthy, asymptomatic lifestyle after stressful events [2]. As much as resiliency involves coping, resilience can also affect profound personal growth and “bouncing back” from these difficult experiences [9].

The prevalence of low resilience has been discussed among the general population and other disaster victims, and this prevalence may increase due to natural disasters. For example, among direct victims, the prevalence of PTSD ranged between 30% and 40% [1]. These variations in prevalence may be attributed to several factors.

Many sociodemographic and clinical factors have been identified as predictors of the likelihood of longer-term mental health impacts of disasters such as flooding [10]. These include individual resiliency, the severity of the disaster, healthy or maladaptive coping skills, degree of victim involvement, sex, and age [10]. For example, women tend to suffer the psychological effect of floods. Research explains that women are primarily responsible for the children at home and emotionally invest more at home [11]. On the spectrum of age, children are considered more vulnerable and experience lower resilience to natural disasters [12,13] than adults.

Studies have explored the effects of flooding on common mental disorders from high or middle-income countries, and results revealed a significant increase in depression, anxiety, and psychological distress among adults who experience flooding in these countries [3,4,13].

Disasters affect victims’ functioning on both physical and psychological levels [14]; flooding is not exempt. A study in Carlisle, after the flooding, shows several people reported suffering from anxiety and stress, which reduce resilience [3,14,15]. Along with losing physical possessions, disaster survivors are likely to develop some form of instability due to the stressors of a disaster, and resilience is compromised [14,16]. Preexisting psychological disorders may predispose flood victims to a worse condition or vice versa. Future research needs to focus on clinical predictors influencing psychological distress following flooding.

Some predisposing factors make people who experience natural disasters more prone to mental health conditions [3,10,17]. For example, a survey showed that flooding was actively correlated with poor mental health and resiliency and strongly associated with lower psychological distress [18]. While some studies associate mental health repercussions with post-disasters [4], few suggest that people adjust well during a disaster such as flooding [13], and support after post-flooding is protective against psychological impact. In post-disaster situations, community social capital networks provide direct aid, information, financial resources, and emotional and psychological support, and individuals who benefit from this support exhibit high to moderate resilience [13,19].

Previous studies did not compare the effects of flooding and factors contributing to low resilience. However, although the number of studies on the impact of natural disasters on mental health is on the rise, more research is needed to determine predictors of low resilience aside from flood-related variables to improve quality of life and achieve normal to high resilience. This study aims to assess the sociodemographic and clinical predictors of low resilience during the Fort McMurray flooding in 2020. The study examines the sociodemographic and clinical characteristics rather than flood exposure variables related to low resilience in residents of Fort McMurray one year after a devastating flood.

## 2. Methodology

### 2.1. Study Setting

Fort McMurray is in the Northern Alberta Regional Municipality of Wood Buffalo, Alberta, Canada, and is surrounded by boreal forest. The Municipality population has changed significantly over the past two decades—increasing from 51,406 people in 2000 to 111,687 in 2018 [20].

A substantial proportion of the inhabitants of Fort McMurray are employed in the nearby oil sands. Accordingly, the largest population cohort in Fort McMurray is the 30–34 age group, which accounts for 12.3% of the total population. The subsequent largest population cohorts are the 35–39 and 25–29 age groups [21]. Overall, there are more males (54.9%) than females (45.1%) in the population [20].

On 26 April 2020, the Regional Municipality of Wood Buffalo (RMWB) declared a State of Local Emergency (SOLE) due to high water levels and flooding along the Athabasca and Clearwater Rivers. More than 13,000 people were evacuated. The flood was the first large disaster in Canada during the COVID-19 pandemic [21]. After the population of Alberta went into lockdown and people were told to remain at home unless they were obtaining vital services, a large group of residents were forced out of their homes. Due to enforced social isolation, this was both complicated and stressful.

### 2.2. Study Design and Institutional Review Board Approval

A cross-sectional survey was used in this study of data amassed between 24 April and 2 June 2021. Quantitative data was collected via REDCap [22] using online questionnaires. They were distributed via email and randomly sent to residents of Fort McMurray to fill out online. This was made possible by government, school, occupational, and community platforms. An anticipated sample size of 249 was predetermined, with 186 responses to the survey. There was no gross incompletion, yielding a response rate of 74.7%. Prospective participants were provided information about the survey, and consent was obtained from those agreeing to participate. The University of Alberta Health Research Ethics Committee approved the study (Pro00066054).

### 2.3. Data Collection and Outcome Measures

Fort McMurray residents completed the survey forms online at their convenience. Respondents’ demographic characteristics, clinical background, flood exposure experience, and various supports received were gathered using a data collection form designed for this purpose.

Selected predictive factors included in the data collection form were chosen based on a literature review of the factors previously investigated with mental health effects (depression and anxiety), resilience, and other natural disasters [3,23,24]. These variables included age, employment, relationship status, housing status, where residents lived before the flood, respondents lived after the flood, and properties destroyed. Other variables included whether respondents watched devastating damages caused by the flood on media and whether they were fearful for their lives and those of their family and friends during the flood. Clinical variables included a history of mental health problems and previous psychotropic medication use. Finally, variables assessing the level of support received during the flood included insurance companies, the Red Cross, the Government of Alberta, whether residents received counselling after the flood, and whether they would like mental health counselling.

The brief resilience scale (BRS) assesses an individual’s ability to bounce back or recover from stress [25]. The BRS consists of six items measured on a five Likert scale. Responses range from a score of 6 to 30, and the average of the score is recorded. A mean score ranging from 1.00–2.99 indicates low resilience, a score from 3.00–4.30 shows normal resilience, and 4.31–5.00 indicates high resilience [26]. For analysis purposes considering a binary logistic regression, the scores were recategorized into two groups: normal to high resilience and low resilience. This was interpreted based on the following criteria: an average score of less than three (<3) indicates low resilience and more than three (>3) reflects normal to high resilience.

Regarding reliability and validity, the literature shows that the BRS has good internal consistency, with Cronbach alphas ranging from 0.80 to 0.90 and test-retest reliability coefficients for a two-week interval were fair (0.61 to 0.69) [25].

### 2.4. Sample Size Estimation

In accordance with the 2018 census, Fort McMurray has 11,687 residents. Hence, using a 95% confidence interval, and a ±3% margin of error, the sample size needed for prevalence estimates for low resilience to mental disorders will be 1049.

### 2.5. Statistical Analysis

Data were analyzed using SPSS Version 25 [27]. Results of the descriptive analysis were presented as absolute numbers and percentages against gender for all the demographic, clinical, and flood-related variables. A univariate analysis with chi-squared tests was used to ascertain the relationship between each predictor and the likelihood that respondents had high to normal and low resilience. The chi-Square test was used to assess the presence of significant associations between the predictor variables and low resilience. A two-tailed *p*-value of ≤0.05 was deemed significant in the univariate analysis. In the univariate analysis, variables which were significant or near significant were entered into a logistic regression model. Correlation analysis was initially performed to exclude variables with high correlation (Spearman’s correlation coefficient of 0.7 to 1.0 or −0.7 to −1.0) with the regression model. Odds ratios from the binary logistic regression analysis were studied to ascertain the association between the variables in the model and the likelihood of respondents presenting with low resilience, controlling for the other variables in the model. There was no data imputation for missing data either in the univariate analysis or logistic regression analysis.

## 3. Results

Overall, 249 residents accessed the online survey, out of which 186 were completed, yielding a response rate of 74.7%.

### 3.1. Descriptive Sample Characteristics

From Table 1, the predominant age was 26 years or older (93%), females (67%), employed (91%; of which 50% was school board staff), and married, partnered, or cohabiting (71%). Most participants resided in Fort McMurray during the flood (95%) and 82% in the flood areas. Most respondents (78%) owned their house. Notwithstanding, most responders (89%) reported no significant property loss due to the flood, whereas 12.9% reported some minor damage. Regarding media exposure, most respondents reported daily exposure to television images (67%) and newspaper/internet (76%) coverage of the flood and its effects, and 29.3% feared for their lives or their family members’ lives. Regarding clinical history, almost half of the participants reported the absence of a mental health condition before the flooding (48%). Some participants (39%) have sought mental health counselling in the past, whereas 53% reported they would like to receive mental health counselling. The flood did not impact most participants; therefore, they did not receive any support from the Red Cross (79%), the Government of Alberta (79%), and insurers (84%). However, most reported receiving absolute support from family and friends (44%). Table 1 also shows that our respondents’ low resilience prevalence was 37.4%.

### 3.2. Chi-Square/Fisher’s Exact Test of Association between Predictor Variables and Resilience

The chi-square analysis (see Table 2) shows significant associations (*p* < 0.05) between some variables and resilience; age, employment status, history of mental health diagnosis (depression/anxiety), medication for mental health concerns, receiving antidepressants, mental health counselling, and willingness to receive mental health counselling.

### 3.3. Logistic Regression Analysis of Predictors of Low Resilience

Nine of the variables in Table 2 with significant *p* values (*p* < 0.05) or *p*-values that were trending or approaching significance *p* values (0.05 ≤ *p* ≤ 0.1) were entered into the logistic regression model. However, because the variables “support from Red Cross after the flood” and “receiving antidepressants before the flood” were highly correlated (r ≥ 0.7) with other variables, they were not included in the model. The entire model, including all nine predictors, was statistically significant, x^2^ (14, N = 168) = 77.54, *p* < 0.001, which suggested that the model could distinguish between respondents who reported low resilience and those reporting high to moderate resilience. The model explained the variance between 37% (Cox and Snell R^2^) and 50.4% (Nagelkerke R^2^) and correctly classified 76.2% of all cases.

As illustrated in Table 3, six independent variables (age, history of depression, history of anxiety, desire to receive mental health counselling, place of residence before the flood, and support from the Government of Alberta) made unique statistically significant contributions to the model. The strongest predictor of low resilience was participants who would like to receive mental health counselling (Wald = 14.42), (OR = 0.134; 95% CI 0.047–0.378). Results suggest that respondents who would like to receive mental health counselling were about 7.5 times more likely to show low resilience than those who would not want to receive mental health counselling.

Participants diagnosed with depression were 3.9 times more likely to have low resilience than those without a historical diagnosis of depression (OR = 0.258; 95% CI 0.089–0.744). Similarly, those with a history of anxiety are 4.7 times more likely to show low resilience after the flood (OR = 0.212; CI 95% 0.068–0.661) than those with no history of anxiety.

Age was statistically significant. Respondents 25 years and below were about 18.5 times more likely to show low resilience than respondents between the ages of 26 and 40 (OR = 0.054; 95% CI 0.005–0.567). Moreover, respondents under 25 years were nearly 26 times more likely to show low resilience than respondents 40 years and above (OR = 0.038; 95% CI 0.004–0.384). Participants still residing in the same house as before the flood are 10.5 times more likely to show low resilience than those who have relocated, although their relocation is not due to the flood (OR = 0.095; 95% CI 0.021–0.427).

Finally, participants who received some support from the Government of Alberta (OR = 208.343; 95% CI 3.284–13,218.663) were more likely to express low resilience than those who received absolute support from the Government of Alberta. Similarly, participants who did not receive governmental support were more likely to express low resilience than those who received absolute support (OR = 67.688; 95% CI 1.723–2659.09).

## 4. Discussion

The study’s primary goal was to examine the prevalence of low resilience in the aftermath of the 2020 flooding in Fort McMurray and to explore any association between flood experience and resilience. The prevalence of low resilience among our respondents was 37.4%, which is higher than in other studies following a natural disaster. For example, a study conducted two to five months after Hurricane Ike reported that the prevalence of any mental health condition was reduced from 20.6% to 10.9% [28]. People exhibit normal to high resilience and improved mental health after a natural disaster, with 79% bouncing back after tough times [13,29]. Normal to high resilience after a natural disaster may be attributed to many factors, including the support received.

Previous studies agree with our finding, which suggested that increased social support protects against mental health effects, increases resilience, and improves coping post-natural disasters [30,31,32]. Low levels of social support are also associated with post-disaster psychological symptoms and mental health disorders [13]. Furthermore, receiving absolute social support is protective against psychological distress following flooding and other natural disasters [31]. However, community, social, and governmental support play a vital role in resiliency. For example, a study conducted in Japan reported that individuals who received emotional and instrumental support from the community prior to the disaster were less likely to express low resilience to mental health conditions [33]. Hence, community level social cohesion prior to a natural disaster is correlated with reduced risks of PTSD symptoms and increases one’s resilience [34]. In addition, a longitudinal evaluation in a rural community in northern China after an earthquake revealed that the sample that received more support showed a general improvement in post-disaster well-being from 3 to 9 months post-disaster [35]. However, introducing societal interventions will promote resilience in a large population of individuals physically and emotionally [30,36].

A study reported that lower social support was associated with higher post-disaster psychological distress, implying low resilience [3,37] and communal coping is protective against the mental health effects of the trauma associated with natural disasters [38]. Similarly, a meta-analysis of risk factors associated with depression in 31 natural disaster publications established that social support (ORs = 0.95 for adults and 0.21 for children) may protect against mental-health-related stress and maximize resilience [39]. Interestingly, our study has revealed that support from the Government was a protective factor against low resilience after flooding in the logistic regression model. They indicated that individuals who received support from the Government of Alberta were less likely to experience low resilience compared to those who did not receive any support.

### Covariates and Potential Effect Modifiers

Covariates and potential effect modifiers identified in our study are age, pre-existing diagnoses of depression/anxiety, counselling after the flood, and place of residence. There was an association between low resilience and the presence of these variables, ranging from nearly fourfold (history of depression) to an almost twenty-six-fold (age; 40 years and older) less likely to experience low resilience. The rates of low resilience were 77.8% and 40.6% for those ages 25 and younger and for those ages 26 to 40 years (*p* = 0.015), respectively. The more one ages and experiences traumatic events such as natural disasters, the more resilient one become. Older people become more resilient than younger people, who typically have experienced fewer of these kinds of events [4]. Another study also suggests that older individuals may cope better under highly adverse circumstances because of higher resilience and more prosperous life experience [40]. Moreover, younger people exhibit acute psychological impairments and are considered more vulnerable, with minimal resiliency outcomes than older adults, following natural disasters [13,41].

Numerous studies have found that pre-existing distress increases psychopathology and reduces resilience after natural disasters [12]. Published reports indicate that an existing anxiety disorder reduces resilience post-disaster and makes respondents prone to other mental health illnesses, such as post-traumatic conditions [3,17,30]. One study that examined the association between resilience and various socio-contextual factors revealed that depression predicts low resilience in trauma-exposed individuals [40]. The history of anxiety was a risk factor for low resilience 18 months after the Fort McMurray wildfire [10].

Results in our study revealed that respondents who reported a history of depression were more likely to express low resilience after a flood. Depression symptoms may reduce one’s resilience after a natural disaster. For example, a study reported similar finding suggesting that resilience was associated with depression among adolescent survivors of the Wenchuan earthquake [7]. Similarly, a meta-analysis suggested that individuals with depression symptoms report lower levels of trait resilience, whereas others concluded that high resilience reduces mental health [42].

Our findings are also consistent with other mental health studies regarding other disasters, which reported low resilience predictors related to pre-existing mental health conditions and exposure-related variables [37,43,44,45].

Furthermore, our study showed an association between the increased likelihood of low resilience and the desire to receive counselling after the flood. This may stipulate those individuals experiencing low resilience were more likely to seek counselling services. On the contrary, some studies suggest that counselling after a traumatic event may not benefit the individual [46]. When a single session occurs, interventions may not contribute to long-term resilience [47]. The most conservative explanation for this finding is that distressed adults (lowest resilience) are most likely to seek mental health counselling.

Our findings in this study overlap significantly with other reports. Participants who do not reside in the same house as they did before the flood are about eleven-fold less likely to show low resilience compared to those staying at the same home. This result is in relation to another study, which revealed that women who relocated after the earthquake at Gyumri had significantly higher depression scores and lower resilience than women who stayed in the earthquake city [48]. It is likely that remaining at the disaster site is usually associated with high to normal resilience, quicker recovery, and healing [49]. This finding suggests that those who move out of the disaster zone do so because they might not have the resilience to overcome psychological disturbances. On the contrary, children who relocated after an earthquake and children who remained in the earthquake zone had no significant reduction in behavioural difficulties, depression, and post-traumatic disorders [50].

Despite the frequency of post-disaster relocation and evidence of its effect on psychological well-being, there is a relative scarcity of studies; hence, further research is needed in that area. Restoring devastated communities may be difficult when most residents with low resilience suffer from other mental health comorbidities [51]. Moreover, the literature has shown that mental health issues are predominant in disaster communities [52]. Hence, effective tools must be developed to identify vulnerable individuals who need mental health resources.

However, it is essential to study resilience post-disaster and screen residents for depression and anxiety, among other mental health conditions since their existence predicts low resilience. Good governance may be the most critical factor influencing the effectiveness of emergency preparedness, response, and recovery [53]. This has led some researchers to recommend that healthcare systems, government bodies, and policymakers put supportive measures in place post-disaster to increase resilience and reduce mental health effects [13].

## 5. Limitations of This Study

Consideration needs to be made when interpreting our findings. Firstly, we relied on online convenience sampling methods, as the pandemic circumstances limited the systematic approach to data gathering at the population level. Secondly, the study sample size was one hundred and eighty-six which is not fully representative of the community of Fort McMurray with our expected sample size of 1049, hence, increasing the margin of error for our prevalence estimates from the anticipated 3% to about 7%.

Thirdly, we did not try to equate the genders in absolute numbers. It certainly would have been better to have an equal number of males and females, but this strain of research is restrictive in that sense. Lastly, most residents in the disaster zone do not return to the former house post-disaster, thereby decreasing the sample’s population [54]. Most residents severely affected might have chosen not to return to the town within the year when this study was conducted, perhaps permanently. This is likely due to the recent economic downturn in the oil industry, coupled with the COVID-19 global pandemic and the relative isolation of this small town. Regardless of these limitations, the current results add to other natural disaster studies and suggest that support received, clinical variables, and sociodemographics affect resiliency rather than flood exposure variables.

## 6. Conclusions

The study’s results suggest clinical characteristics contribute to low resilience after a natural disaster. Specifically, being younger, having a pre-existing anxiety disorder, having pre-existing depression, and relocation contribute to low resilience after the flood. At the same time, governmental support is protective against low resilience after the flood. Our findings are broadly in accord with the natural disaster and flood-related literature. Further studies are needed to unravel other clinical variables and support contributing to low resilience in flood victims. Further study is recommended to investigate the association between support from a government body and its effect on resilience and the interaction of low resilience and relocation after a flood.

Psychological first aid has become the choice of post-disaster intervention to provide safety and necessities for victims’ post-disaster by promoting adaptive coping, reducing acute stress, and increasing resilience [4]. In addition, policies and post-disaster interventions must be developed to help mitigate future psychopathology and provide overall mental health resilience. Economical and effective mobile health programs, such as daily supportive text messaging, can reduce psychological distress after traumatic events such as floods and improve the resilience of the residents [54,55,56,57,58,59,60]. Our study contributes to the growing body of literature suggesting that mental health promotion is of key importance in the aftermath of natural disasters, as is adequate governmental and social support.

## Figures and Tables

**Table 1:** Descriptive Characteristics of the Sample.

Variables	Male	Female	Total
	n (%)	n (%)	n (%)
**Age (years)**			
≤25	4 (14.8)	9 (5.7)	13 (7.0)
26–40	5 (18.5)	70 (44.0)	75 (40.3)
>40	18 (66.7)	80 (50.3)	98 (52.7)
**Employment status**			
Employed	24 (88.9)	151 (95)	175 (94.1)
Unemployed	3 (11.1)	8 (5.0)	11 (5.9)
**Place of employment**			
School boards	8 (33.3)	79 (52.7)	87 (50.0)
Healthcare industry	0 (0.0)	10 (6.7)	10 (5.7)
Keyano College	1 (4.2)	19 (12.7)	20 (11.5)
Oil sands industry	6 (25.0)	7 (4.7)	13 (7.5)
Municipal or government agency	5 (20.8)	8 (5.3)	13 (7.5)
Other	4 (16.7%)	27 (18.0)	31 (17.8)
**Marital status**			
Married/partnered/cohabiting	17 (63.0)	115 (72.3)	132 (71.0)
Divorced/separated/widowed	1 (3.7)	17 (10.7)	18 (9.7)
Single	9 (33.3)	27 (17.0)	36 (19.4)
**Did respondents reside in Fort McMurray during the 2020 flood?**			
Yes	25 (92.6)	151 (95.0)	176 (94.6)
No	2 (7.4)	8 (5.0)	10 (5.4)
**Affected residence during the 2020 flood**			
No flooding areas	20 (80.0)	125 (82.8)	145 (82.4)
Flooding areas	5 (20.0)	26 (17.2)	31 (17.6)
**Homeownership prior to 2020 Fort McMurray flooding?**			
Own home	17 (63.0)	124 (78.0)	141 (75.8)
Renting	10 (37.0)	35 (22.0)	45 (24.2)
**Current homeownership**			
Own home	18 (66.7)	127 (79.9)	145 (78.0)
Renting	9 (33.3)	32 (20.1)	41 (22.0)
**History of mental health diagnosis from a health professional**			
Depression	6 (22.2)	52 (32.7)	58 (31.2)
Bipolar Disorder	1 (3.7)	5 (3.1)	6 (3.2)
Anxiety	9 (33.3)	69 (43.4)	78 (41.9)
Schizophrenia	0 (0.0)	0 (0.0)	0 (0.0)
Personality Disorder	0 (0.0)	2 (1.3)	2 (1.1)
Other	5 (18.5)	12 (7.5)	17 (9.1)
No mental health diagnosis	15 (55.6)	75 (47.2)	90 (48.4)
**History of psychotropic medications**			
Not on psychotropic medication	9 (33.3)	57 (35.8)	66 (35.5)
**Respondents received MH counselling in the past year**			
	9 (33.3)	63 (39.6)	72 (38.7)
**Respondents would like to receive MH counselling**	9 (33.3)	89 (56.0)	98 (52.7)
**Where did respondents live just prior to the 2020 Fort McMurray flooding?**			
Fort McMurray	22 (91.7)	145 (96.7)	167 (96.0)
Other areas	2 (8.3)	5 (3.3)	7 (4.0)
**Respondents witnessed the flooding of homes or structures in Fort McMurray?**	20 (83.3)	111 (74.0)	131 (75.3)
**Respondents who were fearful for their life or the lives of their friends or family during the flooding?**	5 (20.8)	46 (30.7)	51 (29.3)
**During the 2020 Fort McMurray flooding, how frequently did respondents watch television images about the devastation caused by the floods?**			
Daily	15 (62.5)	101 (67.3)	116 (66.7)
Less than daily	6 (25.0)	31 (20.7)	37 (21.3)
Not at all	3 (12.5)	18 (12.0)	21 (12.1)
**During the 2020 Fort McMurray flooding, how frequently did you read newspaper and internet articles related to the devastation caused by flooding?**			
Daily	15 (62.5)	117 (78.5)	132 (76.3)
Less than daily	8 (33.3)	27 (18.1)	35 (20.2)
Not at all	1 (4.2)	5 (3.4)	6 (3.5)
**Property loss because of the floods in Fort McMurray:**			
The house was completely destroyed	0 (0.0)	0 (0.0)	0 (0.0)
The house suffered substantial damage	1 (3.7)	8 (5.0)	9 (4.8)
The house suffered slight damage	1 (3.7)	4 (2.5)	5 (2.7)
The car was completely destroyed	1 (3.7)	3 (1.9)	4 (2.2)
The business was completely destroyed	0 (0.0)	6 (3.8)	6 (3.2)
No loss	24 (88.9)	141 (88.7)	165 (88.7)
Live in the same house you lived in before the floods	18 (75.0)	129 (86.6)	147 (85.0)
Yes	5 (20.8)	17 (11.4)	22 (12.7)
No; I live in a different house even though my previous home was not destroyed by the flood			
No; I live in a different house because my previous home was destroyed by the flood	1 (4.2)	3 (2.0)	4 (2.3)
**Received sufficient support from family and friends during and after the floods**			
Yes, absolute support	10 (43.5)	64 (44.4)	74 (44.3)
Yes, some support	3 (13.0)	23 (16.0)	26 (15.6)
Yes, but only limited support	2 (8.7)	15 (10.4)	17 (10.2)
Not at all	8 (34.8)	42 (29.2)	50 (29.9)
**Received sufficient support from Red Cross**			
Yes, absolute support	2 (8.3)	11 (7.5)	13 (7.6)
Yes, some support	2 (8.3)	4 (2.7)	6 (3.5)
Yes, but only limited support	0 (0.0)	7 (4.8)	7 (4.1)
Not at all	2 (8.3)	8 (5.4)	10 (5.8)
Not Applicable as I was not impacted by the floods	18 (75.0)	117 (79.6)	135 (78.9)
**Received sufficient support from the Government of Alberta**			
Yes, absolute support	2 (8.3)	9 (6.1)	11 (6.4)
Yes, some support	2 (8.3)	5 (3.4)	7 (4.1)
Yes, but only limited support	0 (0.0)	6 (4.1)	6 (3.5)
Not at all	2 (8.3)	10 (6.8)	12 (7.0)
Not Applicable as I was not impacted by the floods	18 (75.0)	117 (79.6)	135 (78.9)
**Received sufficient support from insurers**			
Yes, absolute support	0 (0.0)	5 (3.4)	5 (2.9)
Yes, some support	1 (4.2)	4 (2.7)	5 (2.9)
Yes, but only limited support	1 (4.2)	6 (4.1)	7 (4.1)
Not at all	1 (4.2)	10 (6.8)	11 (6.4)
Not Applicable as I was not impacted by the floods	21 (87.5)	122 (83.0)	143 (83.6)
**Resilience**			
Low resilience	6 (25)	58 (39.5)	64 (37.4)
High resilience	18 (75)	89 (60.5)	107 (62.6)

**Table 2:** Chi-Square Analysis/Fisher’s Exact test * of association between variables and resilience.

Variables	Low Resilience	High to Normal	Chi-Square/Fisher’s Exact Test * Value	*p*-Value
		Resilience		
**Age (years)**				
≤25	7 (77.8%)	2 (22.2%)	8.1	0.015
26–40	28 (40.6%)	41 (59.4%)		
>40	29 (31.2%)	64 (68.8%)		
**Employment status**				
Employed	56 (34.8%)	105 (65.2%)	8.22	0.006
Unemployed	8 (80.0%)	2 (20.0%)		
**Place of employment**				
School boards	27 (34.6%)	51 (65.4%)	3.454 *	0.642
Healthcare industry	3 (33.3%)	6 (66.7%)		
Keyano college	6 (30.0%)	14 (70.0%)		
Oil Sands industry	5 (41.7%)	7 (58.3%)		
Municipal or Government Agency	2 (16.7%)	10 (83.3%)		
Other	13 (44.8%)	16 (55.2%)		
**Relationship status**				
Married/Partnered/Cohabiting	45 (36.3%)	79 (63.7%)	1.12	0.634
Divorced/Separated/Widowed	5 (31.3%)	11 (68.8%)		
Single	14 (45.2%)	17 (54.8%)		
**Housing status**				
Own home	54 (39.7%)	82 (60.3%)	1.47	0.247
Renting	10 (28.6%)	25 (71.4%)		
**History of Depression**				
Yes	34 (63.0%)	20 (37.0%)	22	0
No	30 (25.6%)	87 (74.4%)		
**History of Bipolar Disorder**				
Yes	2 (33.3%)	4 (66.7%)	0.04	1
No	62 (37.6%)	103 (62.4%)		
**History of Anxiety**				
Yes	41 (56.9%)	31 (43.1%)	20.2	0
No	23 (23.2%)	76 (76.8%)		
**History of Personality Disorder**				
Yes	0 (0.0%)	1 (100%)	0.6	1
No	64 (37.6%)	106 (62.4%)		
**No history of mental health diagnosis**				
Yes (received MH Dx)	46 (51.7%)	43 (48.3%)	16.1	0
No	18 (22.0%)	64 (78.0%)		
**Received Antidepressants before the flood**				
Yes	30 (55.6%)	24 (44.4%)	11.1	0.001
No	34 (29.1%)	83 (70.9%)		
**Received Antipsychotics before the flood**				
Yes	2 (50.0%)	2 (50.0%)	0.28	0.631
No	62 (37.1%)	105 (62.9%)		
**No medication for mental health concerns**				
Yes	32 (52.5%)	29 (47.5%)	9.15	0.003
No	32 (29.1%)	78 (70.9%)		
**Receive MH counseling in the past**				
Yes	33 (50.8%)	32 (49.2%)	7.97	0.006
No	31 (29.2%)	75 (70.8%)		
**Would like MH counseling**				
Yes	49 (55.1%)	40 (44.9%)	24.6	0
No	15 (18.3%)	67 (81.7%)		
**Residing in McMurray during 2020 flood**				
No	5 (71.4%)	2 (28.6%)	3.6	0.104
Yes	59 (36.0%)	105 (64.0%)		
**Residing area during the 2020 flood**				
No flooding area	49 (36.6%)	85 (63.4%)	0.11	0.838
Flooding area	10 (33.3%)	20 (66.7%)		
**Residence prior to 2020 McMurray flood**				
In Fort McMurray	60 (36.6%)	104 (63.4%)	1.21	0.427
Other	4 (57.1%)	3 (42.9%)		
Witness flooding of homes				
No	15 (35.7%)	27 (64.3%)	0.07	0.856
Yes	49 (38.0%)	80 (62.0%)		
**Fearful of life and lives of family and friends during flood**				
No	41 (33.9%)	80 (66.1%)	2.22	0.165
Yes	23 (46.0%)	27 (54.0%)		
**Frequency watching TV of the flood**				
Daily	44 (38.6%)	70 (61.4%)	0.33	0.863
< Daily	12 (33.3%)	24 (66.7%)		
Never	8 (38.1%)	13 (61.9%)		
**Frequency reading newspapers about the flood**				
Daily	49 (37.7%)	81 (62.3%)	0.47	0.813
< Daily	12 (35.3%)	22 (64.7%)		
Never	3 (50.0%)	3 (50.0%)		
**Lose property due to the flood**				
No loss	57 (38.0%)	93 (62.0%)	0.17	0.679
Yes loss	7 (33.3%)	14 (66.7%)		
**Live in the same house prior to the flood**				
Yes	58 (40.3%)	86 (59.7%)	4.430 *	0.095
No (although home not destroyed by flood)	4 (18.2%)	18 (81.8%)		
No (home destroyed by flood)	2 (50.0%)	2 (50.0%)		
**Family and friends support during/after the flood**				
Absolute support	22 (30.1%)	51 (69.9%)	6.12	0.107
Some support	8 (30.8%)	18 (69.2%)		
Limited support	9 (52.9%)	8 (47.1%)		
Not at all	23 (47.9%)	25 (52.1%)		
**Support from Red Cross during/after the flood**				
Absolute support	3 (23.1%)	10 (76.9%)	7.500 *	0.098
Some support	5 (83.3%)	1 (16.7%)		
Limited support	1 (14.3%)	6 (85.7%)		
Not at all	4 (40.0%)	6 (60.0%)		
N/A	50 (37.9%)	82 (62.1%)		
**Support from the Government of Alberta during/after the flood**				
Absolute support	1 (9.1%)	10 (90.9%)	7.681 *	0.088
Some support	5 (71.4%)	2 (28.6%)		
Limited support	3 (50.0%)	3 (50.0%)		
Not at all	4 (33.3%)	8 (66.7%)		
N/A	4 (33.3%)	8 (66.7%)		
**Support from insurers during/after the flood**				
Absolute support	1 (20.0%)	4 (80.0%)	1.927 *	0.789
Some support	3 (60.0%)	2 (40.0%)		
Limited support	2 (28.6%)	5 (71.4%)		
Not at all	4 (36.4%)	7 (63.6%)		
N/A	53 (37.5%)	87 (62.1%)		

* Fisher’s exact test was used when the number of counts in any cell was less than 5.

**Table 3:** Chi-square test of association between demographic and clinical antecedents and the likelihood of low resilience.

Variables		Coefficient	Standard Error	Wald Statistic	*p* Value	Odds Ratio	95% C.I. for Odds Ratio	
							Lower	Upper
**Age (Years)**	≤25			7.805	0.020			
	26–40	−2.924	1.203	5.911	0.015	0.054	0.005	0.567
	≥40	−3.277	1.183	7.671	0.006	0.038	0.004	0.384
**Employment status**		−1.609	1.186	1.840	0.175	0.200	0.020	2.046
**Depression (No)**		−1.355	0.540	6.282	0.012	0.258	0.089	0.744
**Anxiety (No)**		−1.552	0.581	7.140	0.008	0.212	0.068	0.661
**Not on any medication for mental health concerns**		0.327	0.599	0.298	0.585	1.387	0.429	4.487
**Received mental health counselling in the past year**		1.039	0.558	3.466	0.063	2.827	.947	8.445
**Would like to receive mental health counselling**		−2.010	0.529	14.421	0.000	0.134	0.047	0.378
**Residing in the same house before the flood**	Yes			9.458	0.009			
	No, although not due to flooding	−2.358	0.769	9.401	0.002	0.095	0.021	0.427
	No, due to flooding	0.141	1.325	0.011	0.915	1.151	0.086	15.439
**Receiving Support from the Government of Alberta during and after the floods**	Absolute			7.402	0.116			
	Some support	5.339	2.117	6.358	0.012	208.343	3.284	13,218.663
	Limited support	3.028	1.862	2.643	0.104	20.651	0.537	794.475
	No support	4.215	1.873	5.065	0.024	67.688	1.723	2659.091
	Not applicable	3.278	1.687	3.776	0.052	26.526	0.972	723.931

## Data Availability

Data associated with this study will be made freely available upon reasonable request to the corresponding author.
